# miR-542-5p Attenuates Fibroblast Activation by Targeting Integrin α6 in Silica-Induced Pulmonary Fibrosis

**DOI:** 10.3390/ijms19123717

**Published:** 2018-11-22

**Authors:** Jiali Yuan, Ping Li, Honghong Pan, Yan Li, Qi Xu, Tiantian Xu, Xiaoming Ji, Yi Liu, Wenxi Yao, Lei Han, Chunhui Ni

**Affiliations:** 1 Center for Global Health, School of Public Health, Nanjing Medical University, Nanjing 210029, China; yuanjialigogo@126.com (J.Y.); NJMUliping@163.com (P.L.); pp2366335911@126.com (H.P.); liyan_njmu@163.com (Y.L.); Xuqi9876@126.com (Q.X.); tiantianxutt@163.com (T.X.); jxmnjmu@163.com (X.J.); liuyinjmu@163.com (Y.L.); ywx3737@163.com (W.Y.); 2 The Key Laboratory of Modern Toxicology of Ministry of Education, Nanjing Medical University, Nanjing 210029, China; 3 Institute of Occupational Disease Prevention, Jiangsu Provincial Center for Disease Control and Prevention, Nanjing 210009, China; hanlei@jscdc.cn

**Keywords:** Silicosis, miRNA, integrin, fibroblast, proliferation

## Abstract

Silicosis is a very serious occupational disease and it features pathological manifestations of inflammatory infiltration, excessive proliferation of fibroblasts and massive depositions of the extracellular matrix in the lungs. Recent studies described the roles of a variety of microRNAs (miRNAs) in fibrotic diseases. Here, we aimed to explore the potential mechanism of miR-542-5p in the activation of lung fibroblasts. To induce a pulmonary fibrosis mouse model, silica suspension and the miR-542-5p agomir were administered to mice by intratracheal instillation and tail vein injection. We found that miR-542-5p was significantly decreased in mouse fibrotic lung tissues and up-regulation of miR-542-5p visually attenuated a series of fibrotic lesions, including alveolar structural damage, alveolar interstitial thickening and silica-induced nodule formation. The down-regulation of miR-542-5p was also observed in mouse fibroblast (NIH-3T3) treated with transforming growth factor β1 (TGF-β1). The proliferation and migration ability of NIH-3T3 cells were also inhibited by the transfection of miR-542-5p mimic. Integrin α6 (Itga6), reported as a cell surface protein associated with fibroblast proliferation, was confirmed to be a direct target of miR-542-5p. The knockdown of Itga6 significantly inhibited the phosphorylation of FAK/PI3K/AKT. In conclusion, miR-542-5p has a potential function for reducing the proliferation of fibroblasts and inhibiting silica-induced pulmonary fibrosis, which might be partially realized by directly binding to Itga6. Our data suggested that miR-542-5p might be a new therapeutic target for silicosis or other pulmonary fibrosis.

## 1. Introduction

Silicosis is a progressive, disabling and incurable disease that is characterized by the aberrant proliferation of fibroblasts and the deposition of the extracellular matrix (ECM) [1,2]. Silicosis has been a worldwide problem for over half a century, with increasing incidence and prevalence, especially in developing countries [3,4,5]. China has the highest burden of silicosis, with more than 20,000 new cases reported annually, according to the official website of the Chinese Center for Disease Control and Prevention [5]. Numerous studies over the past few decades have revealed a variety of molecular mechanisms and signaling pathways underlying this disease. As one of the main effector cells, activated pulmonary fibroblasts promote abnormal lung remodeling by secreting a large amount of the extracellular matrix.

MicroRNAs are a class of small, noncoding, single-stranded RNA with 21–23 nucleotides in length and they post-transcriptionally regulate gene expression through base pairing with the 3’-untranslated region (3’-UTR) of target messenger RNAs (mRNAs) [6,7,8,9]. Accumulated studies have screened and identified multiple dysregulated miRNAs in various diseases, including pulmonary fibrosis. It is reported that miR-1343 suppressed the TGF-β signaling pathway to attenuate lung fibrosis by directly targeting the 3′-UTRs of TGFBR1 and TGFBR2 [10]. Our previous study also revealed that miR-449a had an antifibrotic effect on silica-induced lung fibrosis by targeting autophagy-related Bcl2 mRNA [11]. Besides, miR-489 was observed to attenuate inflammation and fibrotic progression by targeting MyD88 and Smad3 simultaneously [12,13]. However, Chen et al. reported that miR-497-5p promoted pulmonary fibrogenesis via the induction of the differentiation of lung resident mesenchymal stem cells into myofibroblasts [14]. All of these studies showed that miRNAs might participate in the occurrence and development of fibrosis in different ways. Further identification of specific miRNAs and a detailed clarification of their potential roles in mediating silicosis are critical for developing efficient therapies.

In this study, we investigated the role of miR-542-5p, specifically in silica-induced pulmonary fibrosis and the activation of pulmonary fibroblasts. miRNA microarray analysis in our previous study has shown a decrease of miR-542-5p in silica-induced mouse lung fibrosis [15]. We established a silica-induced mouse lung fibrosis model and confirmed the down-regulation of miR-542-5p in fibrotic lung tissues. We found that the ectopic expression of miR-542-5p exhibited a promising preventive and therapeutic effect on silica-induced pulmonary fibrosis. Further exploration also revealed an inhibitory effect of miR-542-5p on the proliferation of pulmonary fibroblasts, which might be partially realized by the regulation of the Itga6/FAK/PI3K/AKT signal cascade.

## 2. Results

### 2.1. miR-542-5p is Decreased in Mouse Lung Tissues in a Model. of Silica-Induced Pulmonary Fibrosis

**Figure 1:**
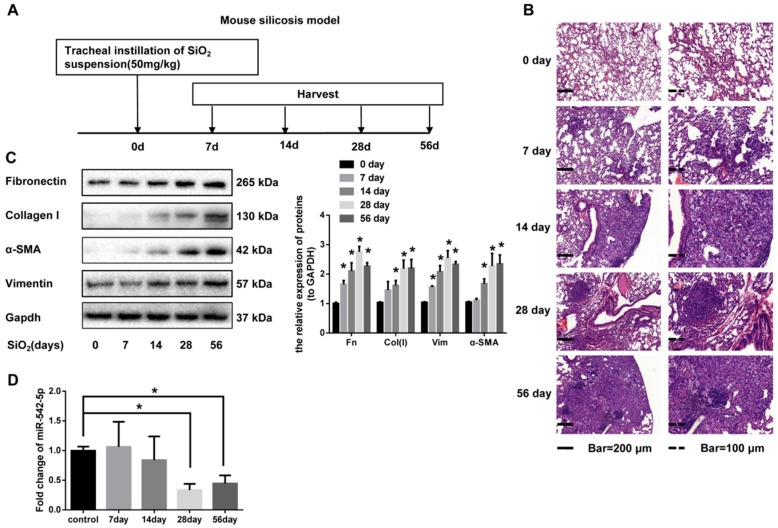
miR-542-5p is down-regulated in the lung tissues of mice with silicosis. (**A**) The mouse model of silicosis. The C57BL/6 mice were tracheally instilled with SiO_2_ suspension (50 mg/kg) and then the tissues were harvested on days 7, 14, 28 and 56 after treatment (*n* = 8 per group). (**B**) The hematoxylin and eosin staining of lung sections (from mice received silica treatment for 0, 7, 14, 28, 56 days) reflected the distribution and severity degree of silicosis and the most representative results were shown. (**C**) Proteins of lung tissues from three randomly selected mice in each group were extracted and Western blot was performed to detect the fibrosis markers (fibronectin, collagen I, α-SMA and vimentin). Fn: fibronectin; Col(I): collagen I; Vim: vimentin. ImageJ software was used to perform a grayscale scan, with semi-quantitating protein bands. GAPDH was used as the interval reference. (**D**) Total RNA of lung tissues (from mice received silica treatment for 0, 7, 14, 28, 56 days) were extracted and the expression level of miR-542-5p was examined by qRT-PCR. U6 was used as the interval reference. Above all, * *p* < 0.05 versus the control group.

To investigate the toxicological effect of silica on lung tissues, we established a mouse silicosis model via a tracheal instillation of silica suspension (Figure 1A). Tissue sections were stained with hematoxylin and eosin (H&E) to exhibit pathological changes. Alveolar wall thickening and inflammatory cell infiltration were observed on day 7 and day 14 after silica instillation. Diffused lung fibrosis and fibrotic nodules appeared on day 28 and day 56 after silica instillation (Figure 1B). Masson staining results indicated a remarkable increase of collagen deposition in the lung tissues of mice treated with silica (Figure 1B). Our data also showed a significant increase of the fibrosis markers (fibronectin, collagen I, vimentin and α-SMA) in a time-dependent manner, which supported the above visible pathological manifestations (Figure 1C, Supplementary file 1, Figure S1). In order to ascertain the changing trend of miR-542-5p in our silicosis mouse model, quantitative real-time polymerase chain reaction (qRT-PCR) analysis was performed and the results displayed markedly decreased the expression of miR-542-5p, with an approximately 67% decline on day 28, compared with the control group (Figure 1D). We also collected information on silicosis patients in the Gene Expression Omnibus (GEO) database and miR-542-5p was reported to be down-regulated in peripheral blood leukocytes form silicosis patients (GSE80555) (Supplementary file 1, Figure S2). The results of our in vitro experiments also showed that the expression of miR-542-5p was significantly decreased in the human fibroblast cell line MRC-5 (Supplementary file 1, Figure S3). Together, these data strongly suggested that miR-542-5p decreased pulmonary fibrosis significantly.

### 2.2. miR-542-5p Attenuates Silica-Induced Pulmonary Fibrosis In Vivo

**Figure 2:**
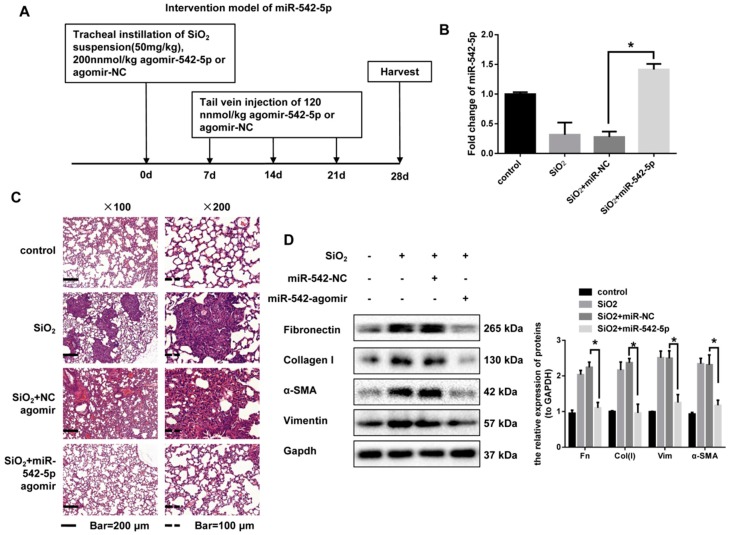
Ectopically expressed miR-542-5p had a therapeutic effect on silica-induced pulmonary fibrosis in mice. (**A**) The mouse model of miR-542-5p overexpression in silica-induced mouse pulmonary fibrosis. The C57BL/6 mice were co-transfected with 200 nmol/kg of either miR-542-5p or miR-NC agomir with 50 mg/kg silica suspension via intratracheal instillation and then injected with 120 nmol/kg miR-542-5p or miR-NC agomir weekly via the tail vein over the next three weeks. Lung tissues were harvested on day 28 (*n* = 8 for each group). (**B**) The expression level of miR-542-5p was increased in the lung tissues treated with miR-542-5p agomir, compared with the group treated with the miR-NC agomir. * *p* < 0.05 versus the SiO_2_ + agomir NC group. (**C**) Sections stained with hematoxylin and eosin of the group treated with silica + miR-542-5p agomir showed a lighter degree of fibrosis compared with the group treated with silica or silica + miR-NC agomir; the most representative results were shown here. (**D**) Protein levels of the fibrosis markers (fibronectin, collagen I, α-SMA and vimentin) of the silica + miR-542-5p agomir group were significantly reversed compared with the silica + miR-NC agomir group (randomly *n* = 3 per group). The gray value of the protein band was quantified by ImageJ and GAPDH was used as an internal reference, * *p* < 0.05 versus the silica + agomir-NC group.

Given that miR-542-5p expression was reduced in fibrotic mouse lung tissues, we next assessed whether increased miR-542-5p was sufficient to prevent the progression of silica-induced fibrosis. To confirm this assumption, an intervention model of miR-542-5p was established in C57BL/6 mice. We co-injected a miR-542-5p agomir or miR-negative control (NC) on day 0 with silica via tracheal instillation and then injected miR-542-5p agomirs or miR-NC via the tail vein on day 7, day 14 and day 21 (Figure 2A). qRT-PCR analysis revealed that, after 28 days of treatment, the miR-542-5p agomir significantly restored the level of miR-542-5p reduced by silica (Figure 2B). Consistent with the pathological changes in Figure 1B, silica treatment also led to a typical inflammatory response, nodule formation and collagen deposition in our prevention model. However, the mice co-injected with miR-542-5p agomir and silica showed attenuated fibrotic pathological manifestations compared to the mice in the silica group (Figure 2C, Supplementary file 1, Figure S4). In addition, the up-regulation of miR-542-5p also reduced the expression level of fibronectin, collagen I, vimentin and α-SMA (Figure 2D). We then scored for pulmonary fibrotic lesions. The severity degree of fibrosis was reduced after miR-542-5p agomir administration in silica + the miR-542-5p agomir group, compared to the silica group, while no significant change was observed in the degree of distribution (Table 1).

**Figure 3:**
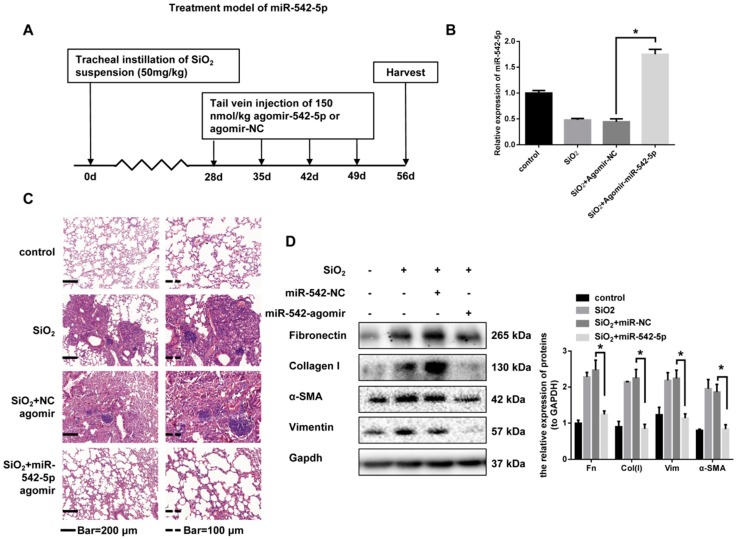
Exogenous miR-542-5p partially reversed silica-induced pulmonary fibrosis in mice. (**A**) The intervention model of miR-542-5p in silica-induced mouse pulmonary fibrosis. The C57BL/6 mice were treated with 50 mg/kg silica suspension via a one-off intratracheal instillation and 140 nmol/kg miR-542-5p agomir or miR-NC agomir was injected to the mouse on days 28, 35, 42 and 49 via the tail vein. Lung tissues were harvested on day 56 (*n* = 8 for each group). (**B**) miR-542-5p expression level in silica + miR-542-5p agomir group was significantly increased, compared with the group treated with silica + miR-NC agomir. * *p* < 0.05 versus the SiO_2_ + agomir NC group. (**C**) Hematoxylin and eosin (H&E) staining of mouse lung tissue showed weakened fibrotic lesions compared with the group treated with silica or silica + miR-NC agomir; the most representative results were shown here. (**D**) Expression levels of the fibrosis markers (fibronectin, collagen I, α-SMA and vimentin) of the silica+miR-542-5p agomir group were significantly reversed, compared with the silica + miR-NC agomir group (randomly *n* = 3 per group). The semi quantification of the protein band was performed by using ImageJ and GAPDH was selected as an internal reference, * *p* < 0.05 versus the silica + agomir-NC group.

To further consider the therapeutic effect of miR-542-5p, we continued to treat mice with a miR-542-5p agomir via tail vein injection from the 28th day after silica treatment. All mice were sacrificed after four weeks (Figure 3A). qRT-PCR analysis confirmed that the miR-542-5p agomir effectively up-regulated miR-542-5p levels in mice (Figure 3B). Pathological sections clearly showed attenuated fibrotic lesions and less fibrotic nodules (Figure 3C, Supplementary file 1, Figure S5). The scoring of the severity and distribution degrees of fibrosis also verified the therapeutic effect of miR-542-5p on established fibrosis (Table 2). The expression of fibrosis markers in miR-542-5p agomir treatment further indicated that mouse lung fibrosis was partially reversed (Figure 3D). All of these demonstrated that miR-542-5p agomir not only played a preventive role in the acute inflammatory phase of fibrosis but also had a promising therapeutic effect after fibrosis development.

### 2.3. miR-542-5p Inhibits the Pro-Fibrotic Effect of TGF-β1 In Vitro

**Figure 4:**
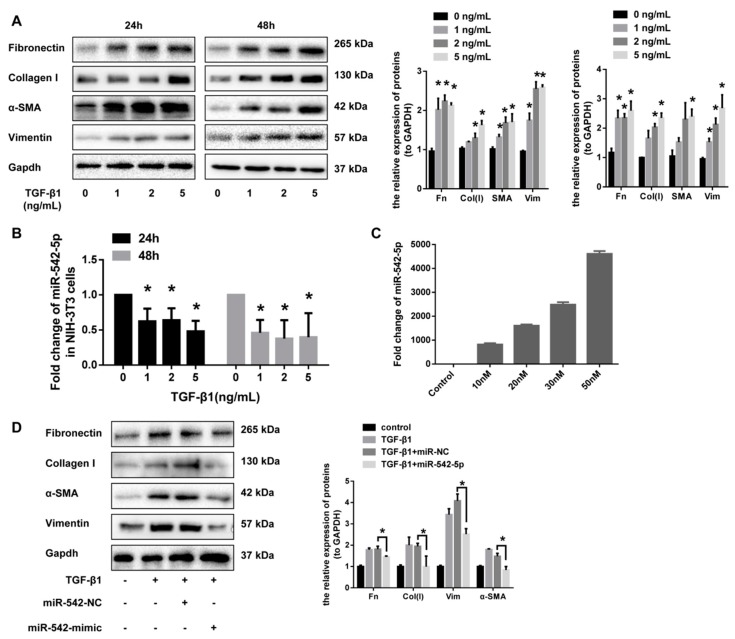
miR-542-5p blocked the activity of NIH-3T3 cells in vitro. (**A**) The protein expression levels of fibronectin, vimentin and α-SMA were obviously elevated in NIH-3T3 cells when treated with an increased dose of TGF-β1 (0, 1, 2, 5 ng/mL) for 24 h or 48 h. The results of Western blot were analyzed by ImageJ software, * *p* < 0.05 versus the control group. (**B**) The miR-542-5p expression levels were significantly decreased in NIH-3T3 cells treated with different doses of TGF-β1 (0, 1, 2, 5 ng/mL) for 24 h or 48 h, * *p* < 0.05 versus the control group. (**C**) miR-542-5p mimics transfection efficiency. Different concentrations (10 nM, 20 nM, 30 nM, 50 nM) of miR-542-5p mimic showed different transfection efficiencies and 30 nM was chosen in this study to optimize the experiment. (**D**) Western blot analysis was performed to detect the fibrosis markers (fibronectin, collagen I, α-SMA and vimentin) in NIH-3T3 cells pretreated with miR-542-5p mimics (30 nM) for 24 h, followed by TGF-β1 (5 ng/mL) for 48 h. * *p* < 0.05 versus the TGF-β1 + mimic-NC group.

Transforming growth factor β1 (TGF-β1) signaling has been well studied as a critical driver of fibrosis progression through the induction of proliferation and the differentiation of pulmonary fibroblasts. The abnormal activation of pulmonary fibroblasts are thought to be a major source of the extracellular matrix that are over-deposited in the lungs. To further validate the effect of miR-542-5p on the activation of lung fibroblasts, we up-regulated miR-542-5p levels in fibroblasts by transfection with miRNA mimics. A dose-dependent increase of fibrosis markers (fibronectin, collagen I, α-SMA and vimentin) in NIH-3T3 cells was observed after 24 and 48 h of TGF-β1 treatment (Figure 4A). miR-542-5p also showed a dose-dependent decrease in NIH-3T3 cells treated in the manner described above (Figure 4B). To investigate whether miR-542-5p had an inhibitory effect on TGF-β1-induced fibroblasts proliferation, NIH-3T3 cells were transfected with different doses of miR-542-5p mimic or control mimic, in order to select a suitable transfection concentration (30 nM) (Figure 4C). As hypothesized, exogenous miR-542-5p showed a remarkable inhibition in TGF-β1-induced fibrotic progress in NIH-3T3 cells. Western blot analysis indicated that the expression of fibronectin, collagen I, vimentin and α-SMA was also decreased in miR-542-5p, plus the TGF-β1 group compared with the TGF-β1 treatment group (Figure 4D).

### 2.4. miR-542-5p Inhibits Proliferation, Migration and Cell. Cycle of NIH-3T3 Cells

**Figure 5:**
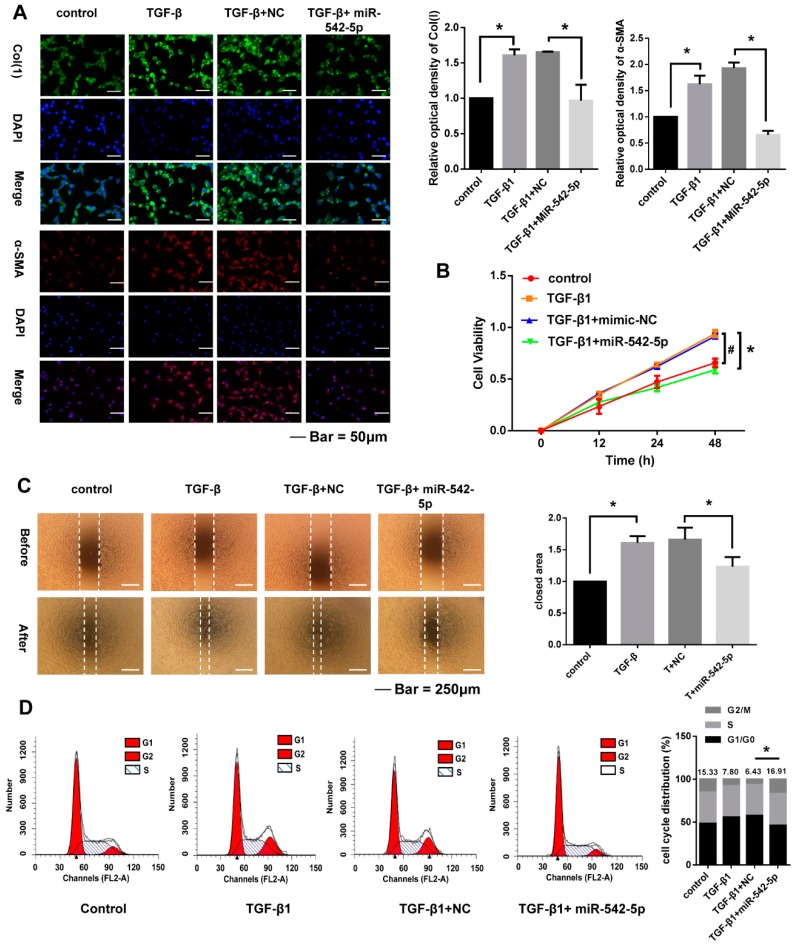
miR-542-5p inhibited the pro-fibrotic effect of TGF-β1 on NIH-3T3 cells. (**A**) The fluorescence intensity of collagen I and α-SMA in NIH-3T3 cells co-treated with 5 ng/mL TGF-β1 and 30 nM miR-542-5p mimic was notably lower than the cells treated with 5 ng/mL TGF-β1; * *p* < 0.05 was considered to be statistically significant. (**B**) The proliferation ability of the NIH-3T3 cells co-treated with 5 ng/mL TGF-β1 and the 30 nM miR-542-5p mimic was significantly reduced compared with the cells treated with 5 ng/mL TGF-β1 48 h after treatment. # TGF-β1+mimic NC group vs. Control group, * TGF-β1+mimic NC group vs. TGF-β1+miR-542-5p group. *p* < 0.05 for # and *. (**C**) The migration ability of NIH-3T3 cells co-treated with 5 ng/mL TGF-β1 and 30 nM miR-542-5p mimic was notably lower than that of the cells treated with 5 ng/mL TGF-β1,* *p* < 0.05 was considered statistically significant. (**D**) Flow cytometric analysis showed that the numbers of TGF-β1 treated cells in S phase was increased remarkably compared to the control group, while the transfection of miR-542-5p mimic restored this increase on the cell cycle, * *p* < 0.05 was considered statistically significant.

To better evaluate the biological role of miR-542-5p expression on the response of NIH-3T3 cells to TGF-β1, we transiently increased intracellular exogenous miR-542-5p by miRNA mimics to detect the alternation of cell proliferation, migration and the cell cycle. The results of the immunofluorescence assays showed that TGF-β1 significantly enhanced the type-1 collagen and α-SMA expression in NIH-3T3 cells, which was then effectively inhibited by miR-542-5p (Figure 5A). Upregulated miR-542-5p also markedly reduced the proliferation and migratory of activated fibroblasts (Figure 5B,C). Cell-cycle assays by flow cytometry indicated that the treatment with miR-542-5p counteracted the S-phase accumulation of NIH-3T3 cells induced by TGF-β1 (Figure 5D).

### 2.5. miR-542-5p Directly Binds to the 3’-UTR of Itga6

**Figure 6:**
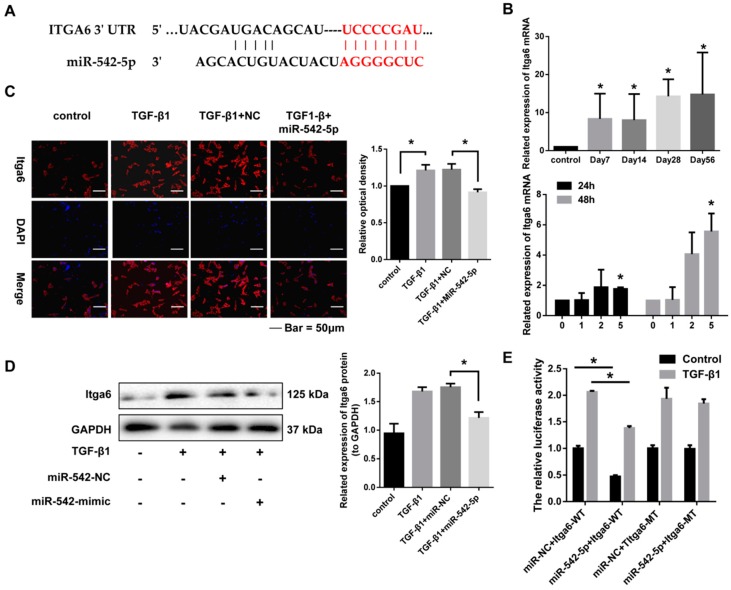
Integrin α6 (Itga6) was a direct target of miR-542-5p. (**A**) Schematic outlining the predicted binding sites for miR-542-5p in the 3’-UTR of Itga6 mRNA. Red part represented the base-pairing of the seed sequence of miR-542-5p and the 3’UTR of Itga6. (**B**) The expression level of Itga6 mRNA was significantly increased in the lung tissues of mice treated with SiO2 on the seventh day, the 14th day, the 28th day and the 56th day and cells treated with different doses of TGF-β1 (0, 1, 2, 5 ng/mL) at 24 h and 48 h, * *p* < 0.05 versus the control group. (**C**) The fluorescence intensity of Itga6 in NIH-3T3 cells co-treated with 5 ng/mL TGF-β1 and 30 nM miR-542-5p mimic was notably lower than the cells treated with 5 ng/mL TGF-β1; The ImageJ software was used for fluorescence quantification, * *p* < 0.05 was considered to be statistically significant. (**D**) The protein level of Itga6 in NIH-3T3 cells co-treated with 5 ng/mL TGF-β1 and 30 nM miR-542-5p mimic was notably lower than the cells treated with 5 ng/mL TGF-β1, * *p* < 0.05 versus the TGF-β1 + mimic-NC group. (**E**) Effects of the miR-542-5p mimic on Itga6 3′-UTR luciferase reporters in NIH-3T3 cells. Luciferase activities were calculated as the ratio of firefly/Renilla activities and normalized to the miR-NC + Itga6 wild type (WT) group. * *p* < 0.05 was considered statistically significant.

miRNAs usually function by binding the 3’-UTR of target genes and then silencing or degrading these mRNAs. Integrin α6 (Itga6) was predicted to be the potential target of miR-542-5p by the targetscan bioinformatics software (Figure 6A). qRT-PCR was then performed to explore the expression relationship between integrin α6 and miR-542-5p. As expected, Itga6 mRNA significantly increased in silica-induced lung tissues and TGF-β1-stimulated fibroblasts, which showed an opposite expression trend to miR-542-5p (Figure 6B,C). Besides, exogenous miR-542-5p significantly reduced the protein level of Itga6 that was up-regulated by TGF-β1 in NIH-3T3 cells (Figure 6D). To verify whether miR-542-5p could bind the 3’-UTR of Itga6, we constructed psi-CHECK2-vector containing a 3’-UTR sequence with mutation or non-mutation on the predicted miR-542-5p site. The luciferase reporter plasmids, together with the miR-542-5p mimic or the control mimic, were transfected into NIH-3T3 cells treated with or without TGF-β1. Our data indicated that although TGF-β1 treated cells showed significant enhancement of the luciferase activity in all groups, the miR-542-5p mimic significantly reduced the luciferase activity in the wild type group, regardless of whether the cells were treated with TGF-β1. However, no remarkable reduction of luciferase activity was observed in the mutation group (Figure 6E). All of the above data demonstrated that Itga6 was a direct target of miR-542-5p.

### 2.6. Itga6 Affects Fibroblast Activation through the FAK Signaling Pathway

**Figure 7:**
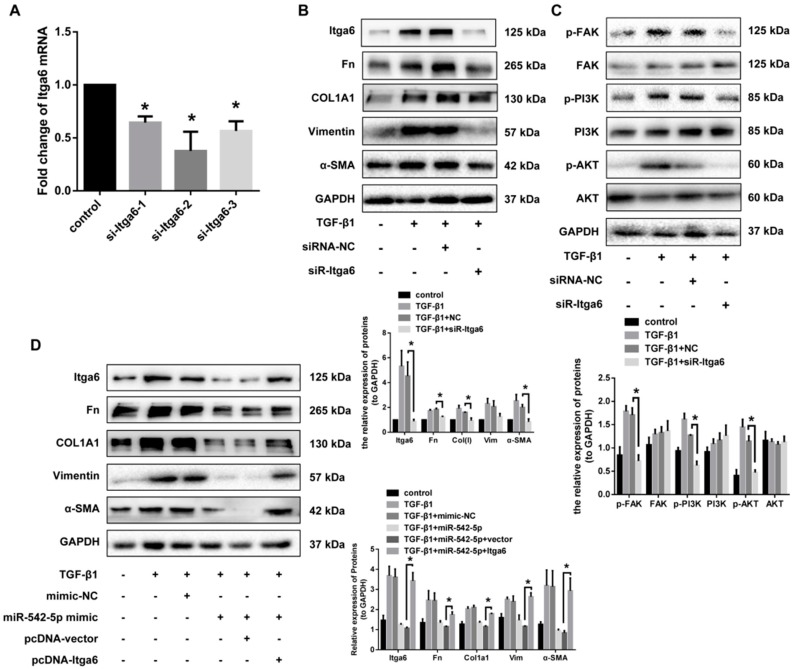
miR-542-5p suppressed fibroblast differentiation by targeting Itga6 through the FAK signaling pathway. (**A**) Knockdown efficiency of three siRNAs towards Itga6. (**B**) The protein level of Itga6 and other fibrosis markers in NIH-3T3 cells transfected with siR-Itga6. Except for the inhibition of Itga6 expression, siR-Itga6 restored the level of fibrosis markers (fibronectin, collagen I, *α-*SMA and vimentin), which were previously increased by TGF-β1. * *p* < 0.05 versus the TGF-β1 + siRNA-NC group. (**C**) Western blot analysis of protein levels of the phosphorylated or non-phosphorylated FAK/PI3K/AKT signaling pathway, * *p* < 0.05 was considered to be statistically significant. (**D**) Western blot analysis of fibrosis markers (fibronectin, collagen I, *α-*SMA and vimentin) in NIH-3T3 cells co-treated with miR-542-5p mimic and pcDNA-Itga6. * *p* < 0.05 versus TGF-β1 plus the miR-542-5p mimic and the pcDNA vector group.

It is reported that fibroblast differentiation induced by TGF-β1 is dependent on cell adhesion and integrin signaling via focal adhesion kinase. Moreover, FAK/PI3K/AKT signaling pathway has long been considered to be an indispensable non-canonical TGF-β signal in fibroblast activation. To determine whether Itga6 had a role in the activation of NIH-3T3, we constructed three siRNAs targeting ITGA6 and the one with the best interference effects was selected to co-treat cells with TGF-β1 (Figure 7A). In addition to the significant down-regulation of Itga6, siR-Itga6 also significantly reduced the protein expression of other fibrosis indicators, including fibronectin, collagen I, vimentin and α-SMA (Figure 7B). Otherwise, FAK/PI3K/AKT signaling pathways were observed to be activated by TGF-β1 in our study, while the phosphorylation level of FAK/PI3K/AKT was significantly inhibited after the knockdown of Itga6, compared to the cells stimulated with TGF-β1 alone (Figure 7C). We then studied the effect of Itga6 on the fibrotic progress of fibroblasts, together with miR-542-5p. TGF-β1-treated 3T3 cells were transfected with pCI-Itga6 and miR-542-5p mimics, separately or together. Upregulation of miR-542-5p in 3T3 cells decreased the fibrosis markers expression as we observed earlier, while the transfection of pCI-Itga6 effectively rescued TGF-β1-induced fibroblast activation (Figure 7D). These results indicated that the inhibition of fibroblast activation by miR-542-5p was dependent, or at least partially dependent, on the regulation of Itga6 expression.

## 3. Discussion

Silica is known as an important occupational hazard that causes silicosis and accumulated evidence showed that silica stimulation can also result in lung cancer over the last several decades. Silicosis is a major type of pneumoconiosis and it is characterized by the destruction of alveolar structure, excessive deposition of the extracellular matrix and aberrant fibroblast proliferation. Although researchers have never stopped studying the pathogenesis of silica disease, lack of effective progress has been achieved in recent years. Small non-coding miRNAs with conserved sequences have entered researchers’ fields of vision as promising therapeutic candidates. The development of miRNA-directed therapy may be a potent strategy to improving the current predicament. In this study, we focused on the expression, regulation and the potential role of miR-542-5p in silicosis.

miR-542-5p was first reported dysregulated in neuroblastoma and its expression levels were very significantly inversely correlated with the amplification of MYCN, a well-known oncogene that is associated with a variety of tumors [16,17,18,19,20]. Subsequent studies indicated that miR-542-5p could also play a role as a tumor suppressor in various cancers by directly binding to EGFR [13,21,22]. However, Cheng et al. reported that miR-542-5p also showed a pro-tumorigenesis effect in human osteosarcomas. HUWE1 was identified to be the direct target of miR-542-5p and the inhibition of HUWE1 promoted the proliferation of osteosarcoma in vitro and in vivo [23,24]. In neuroblastoma cells, the overexpression of miR-542-5p was also associated with the activation of apoptotic pathways in tumor cells [17]. More interestingly, overexpressed miR-542-5p inhibited cell activation and induced a significantly ageing phenotype in human embryonic lung fibroblasts (IMR90) [25]. All of these evidences demonstrated that miR-542-5p appears to be involved in a variety of physiological and pathological processes as an important regulator of cell proliferation. However, the effect of miR-542-5p on fibrotic disorders has not been reported yet. By confirming the decreased expression of miR-542-5p in our previous miRNA microarray expression profile, we further showed that miR-542-5p was significantly decreased in mouse lung tissues exposed to silica dust, as well as in NIH-3T3 cells treated with TGF-β1.

In this study, we selected two forms of exogenous miRNA products (miRNA mimics and miRNA agomirs) to explore the role of miR-542-5p in vitro and in vivo. Although both of them can mimic exogenous miRNAs, the miRNA agomir has a higher stability than the miRNA mimic, which makes it more suitable for in vitro experiments. The fibrosis-suppressive function of miR-542-5p was observed both in our preventive model and the therapeutic model by immunohistochemistry and the detection of fibrotic markers. More deeply, exogenous miR-542-5p markedly inhibited the proliferation of NIH-3T3 cells in vitro, as evidenced by the results of the CCK8 assay, wound healing assay and cell cycle detection. miR-542-5p, causing the inhibition of fibroblast proliferation, may lead, in part, to a block in the progression of fibrosis, thus resulting in a protective effect of miR-542-5p against silica-induced fibrosis.

Integrins are cell surface receptors that play a pivotal role in cell adhesion, migration, invasion, growth and survival. Integrins are heterodimeric proteins that are mainly composed of one α subunit and one β subunit. At least 18 α and eight β subunits are identified to form 24 integrin pairs with different distributions and overlapping specificities. Recently, αvβ3, αvβ5, αvβ6 and αvβ8 integrins have all been shown to theoretically influence the pathogenesis of fibrosis [26,27,28,29,30]. It is reported that the identification of an Arg-Gly-Asp (RGD) sequence in the latency-associated protein (LAP) of TGFβ1, a classic pro-fibrotic cytokine, facilitated the activation of a TGF-β signaling pathway by at least four αv-containing integrins (αvβ3, αvβ5, αvβ6 and αvβ8) in vitro. The role of the αvβ6 integrin has been particularly well described in the pathogenesis of idiopathic pulmonary fibrosis (IPF) in previous studies [31,32,33]. Integrin α6 generally binds to integrin βl/β4 to form heterodimers and it regulates disease progression by altering cellular invasion and migration capabilities [34,35]. Chen et al. reported that α6-integrin was a matrix stiffness-sensitive molecule, which renders an invasive phenotype on fibroblasts and mediates experimental pulmonary fibrosis [36]. Of interest, α6-integrin was predicted and verified as a direct target of miR-542-5p by the TargetScan database and dual luciferase activity assays in our study, respectively. Otherwise, Itga6 was up-regulated in silica-exposed mouse lung tissues and TGF-β1-treated NIH-3T3 cells. The fibrosis markers of fibroblasts were significantly reduced after Itga6 knockdown by siR-Itga6, suggesting that Itga6 might be an important key molecule in the development of fibrosis. Furthermore, the knockdown of Itga6 remarkably attenuated the proliferation and differentiation of fibroblasts into myofibroblasts. The results of function–rescue assays also clarified that the functional role of miR-542-5p in the regulation of pulmonary fibrosis was dependent or partially dependent on the participation of Itga6.

Focal adhesion kinase (FAK) is an integrin-associated signaling molecule that is expressed by many cell types [37,38]. FAK has been reported to be a crucial factor in promoting cell survival and growth downstream of ECM-induced integrin signaling and endocytosis-defective integrin mutants reduced integrin-mediated pFAK-Y397, pAkt-S473 and pErk1/2 signaling [39]. In addition, the FAK/PI3K/AKT signaling cascade is thought to be an important pathway in pulmonary fibrosis and it is involved in the activation of a variety of vital downstream signaling molecules [23,40,41,42,43,44]. Dynamic changes to the matrix composition and stiffness lead to the activation of FAK signaling and enhance the response of fibroblasts to TGF-β1 [36]. Physiological stretches at specific intensities induced the proliferation of human urothelial cells through integrin α6-FAK signaling pathways [45]. Similarly, this Itga6-FAK/PI3K/AKT signal cascade reaction was significantly weakened after interference with the expression of Itga6 in this study, which might explain the important signal transduction role of Itga6 in the process of fibrosis.

## 4. Materials and Methods

### 4.1. Ethics Statement

All animal procedures were conducted in accordance with humane animal care standards and all experimental protocols were approved by the Institutional Review Board (IRB) of Nanjing Medical University (NJMU), (China NJMU (2016) 046).

### 4.2. Animal Studies

Male C57BL/6 mice (4–6 weeks of age) used in this study were purchased from the Shanghai Laboratory Animal Center (SLAC, Shanghai, China). For the mouse model of pulmonary fibrosis, C57BL/6 male mice were randomly divided into five groups (*n* = 8 in each group): day 0, 7, 14, 28 and 56 silica groups. The mice were anesthetized using pentobarbital sodium (Dainippon Sumitomo Pharma, Osaka, Japan) through an intraperitoneal injection. The mice were instilled with 50 mg/kg of silica (Sigma Aldrich, St. Louis, MO, USA) in 0.05 mL sterile saline or 0.05 mL sterile saline intratracheally. The mice were sacrificed on day 0, 7, 14, 28 and 56 after instillation. For the inervention model of miR-542-5p, a total of 32 mice were randomly divided into four groups (*n* = 8 in each group): saline, silica, silica plus miR-NC and silica plus miR-542-5p agomir groups. Either 200 nmol/kg miR-542-5p agomir or miR-NC (RiboBio Co., Ltd., Guangzhou, China) was co-administered with silica-suspended saline. Subsequently, 120 nmol/kg miR-542-5p agomir or miR-NC was injected via the tail vein into each mouse weekly. The mice were sacrificed on day 28 following silica administration. For the treatment model of miR-542-5p, the silica-induced pulmonary fibrosis model was established as described above and a total of 150 nmol/kg miR-542-5p agomirs or miR-NC was subjected to each mouse over the next four weeks. The mice were sacrificed on day 56 after silica treatment. The sequence of agomir-miR-542-5p was: cucggggaucaucaugucacaga (double-stranded); the sequence of the miR-NC was: uucuccgaacgugucacgu (double-stranded). Lung tissues were harvested, quick-frozen in liquid nitrogen and stored at −80 °C immediately for further analysis.

### 4.3. Cell. Culture and Treatment

The mouse fibroblasts (NIH-3T3) were purchased from the American Type Culture Collection (ATCC, Manassas, VA, USA) and maintained in Dulbecco’s modified Eagle’s medium (DMEM, Life Technologies/Gibco, Grand Island, NY, USA) containing 10% fetal calf serum (FCS, Life Technologies/Gibco, Grand Island, NY, USA), 100 U/mL penicillin and 100 µg/mL streptomycin (Life Technologies/Gibco, Gaithersburg, MD, USA). The NIH-3T3 cells cultured in a humidified atmosphere containing 5% CO_2_ at 37 °C. For Western blot or qRT-PCR analysis, the NIH-3T3 cells were plated (2 × 10^5^ cells) into 6-well plates overnight. The fibroblasts were treated with 2 ng/mL TGF-β 1 (Sigma-Aldrich, St. Louis, MO, USA) for 48 h and the total RNA or protein was prepared according to the experimental instructions.

### 4.4. Histopathology

The right lower lung tissues of mouse were infiltrated in formalin solution for 48 h and embedded in paraffin. The sections were then cut into 5 μm thick slices and the histological stainings (hematoxylin and eosin staining, Masson-trichrome staining) were performed accordingly, to assess the degree of fibrosis. A variety of pathological changes occur during fibrosis and such alterations were divided into different grades of severity and distribution. A scoring method for lung fibrosis based on alveolar wall thickening, inflammatory lesions, collagen expression and fibrosis was described in previous reports and performed in this study. For lesion severity: 0 = nothing/zero, 1 = marginal, 2 = slight, 3 = moderate, 4 = severe and 5 = very severe. For lesion distribution: 0 = absent, 1 = rare/occasional (10% of the lung area), 2 = sparse/limited (10–25% of the lung area), 3 = moderate (25–50% of the lung area), 4 = extensive/widespread (50–75% of the lung area) and 5 = very extensive/predominant (over 75% of the lung area).

### 4.5. RNA Isolation and Quantitative Real-Time PCR

Total RNA was extracted using TRIzol reagent (Sigma-Aldrich, T9424, St. Louis, MO, USA), according to the manufacturer’s instructions. The concentration and quality of the RNA were confirmed using a Thermo NanoDrop 2000 spectrophotometer (Waltham, MA, USA). Complementary DNA (cDNA) was reverse-transcribed from total RNA (DNase treated or not) using M-MuLV reverse transcriptase (Fermentas, EP0351, Waltham, MA, USA) and random hexamers (Invitrogen, 48190-011, Waltham, MA, USA). The AceQ^®^ qPCR SYBR^®^ Green Master Mix kit (Vazyme Biotech Co., Ltd., Nanjing, China) was used for amplification. A PCR reaction was performed as follows: 94 °C 5 min, then 35 cycles of 94 °C for 1 min, 50 °C for 1 min and 72 °C for 1 min, followed by a single cycle of 72 °C for 10 min. qRT-PCR analysis was performed by the ABI 7900 Real-Time PCR machine (Waltham, MA, USA). Data were quantified by the 2^−△△Ct^ method. The miRNA primer used in this study was a commercial kit, that is, miRNA quantification: Bulge-loopTM miRNA qRT-PCR Primer Sets (one RT primer and a pair of qPCR primers for each set) specific for miR-542-5p were designed by RiboBio (Guangzhou, China). The primer sequences used in this study were as follows: Itga6: F—TGAAGATGGGCCCTATGAAG, R—CTCTTGGAGCACCAGACACA. GAPDH: F—AGCAGTCCCGTACACTGGCAAAC, R—TCTGTGGTGATGTAAATGTCCTCT. U6: F—CTCGCTTCGGCAGCACATATACT, R—ACGCTTCACGAATTTGCGTGTC.

### 4.6. Western Blotting

The protein in lung tissues was extracted in lysis buffer (M-PER reagent for the cells and T-PER reagent for the tissues, Thermo Scientific, Waltham, MA, USA) and the protein in the cells was extracted with RIPA buffer (50 mM Tris-HCl pH 7.4, 150 mM NaCl, 1% NP40, 0.25% Na-deoxycholate) supplemented with complete protease inhibitor cocktail (Roche, Basel, Switzerland, 04-693-131-001) and 1 mM phenylmethylsulphonyl fluoride (PMSF; Sigma-Aldrich, P7626). A total of 60 µg protein of each sample was electrophoresed on 12.5% polyacrylamide gradient gels and then transferred to nitrocellulose membranes. The membranes were incubated in blocking buffer (5% fat-free milk) prior to incubation with primary antibodies 4 °C overnight. After incubation with the corresponding secondary antibody (Beyotime Bio, Shanghai, China), the protein bands were imaged using a ChemiDoc XRS + imaging system (Bio-Rad Laboratories, Inc., Hercules, CA, USA). The following antibodies were used in the study: anti-fibronectin (Epitomics, Cambridge, UK, 1574-1, 1:1000), anti-GAPDH (Beyotime, Shanghai, China, AG019, 1:1000), anti-collagen I (Abcam, Cambridge, UK, ab34710, 1:5000), anti-α-SMA (Abcam, ab32575, 1:1000, anti-vimentin (Cell Signaling, Danvers, MA, USA, 5741S, 1:1000), anti-Itga6 (Santa Cruz, Dallas, TX, USA, sc-374057, 1:500), anti-FAK (Santa Cruz, sc-1688, 1:500), anti-p-FAK (Santa Cruz, sc-81493, 1:500), anti-PI3K (Cell Signaling, #4257, 1:1000), anti-p-PI3K (Cell Signaling, #4228, 1:1000), anti-AKT (Cell Signaling, #4691, 1:1000) and anti-p-AKT (Cell Signaling, #4060, 1:1000). The densitometric analysis of Western blot and all the immunoblots were showed in Supplementary file 2 and 3.

### 4.7. miRNA Mimic and siRNA Transfection

Cells were cultured to 5 × 10^5^/well in a 6-well cell culture plate for 24 h and then transfected with 30 nM of miRNA mimic (miR-542-5p mimic and control mimic) or 50 nM siRNA (siR-Itga6 or control siRNA) using riboFECT™ CP Reagent (Ribobio Co., Ltd.) according to the manufacturer’s instructions. MicroRNA mimics and siRNAs were synthesized by Ribobio Co., Ltd. (Guangzhou, China).

### 4.8. Wound-Healing Assay

For cell migration assays, cells were seeded in 6-well plates at a density of 7 ×10^5^ per well. The wound was made by passing the plastic tip through a single layer of cells at the bottom of the culture dish. Twenty-four hours after scratching, wound closure was observed and photographed by a phase contrast microscope. The coverage area (%) of migrating cells was determined by the following equation: ((0 h wound area − wound area after 24 h)/0 h wound area) × 100%.

### 4.9. CCK-8 Assay

For cell proliferation assays, a total of 3000 cells were seeded into 96-well plates. After 12 h of culture, cells were given their corresponding treatments. The cell proliferation was determined by the Cell Counting Kit-8 (Beyotime Biotech, Shanghai, China) according to the manufacturer’s protocol. The absorbance at 450 nm of the cells at each time point was used to plot the cell proliferation curves.

### 4.10. Luciferase Assays

For luciferase assays, the plasmid containing the Itga6 3’-UTR and the Itga6 3’-UTR, in which the putative binding sites had been mutated, were constructed by psiCHECK vector and verified by DNA sequencing (Generay Biotech Co., Ltd., Shanghai, China). NIH-3T3 cells were cultured in 24-well plates and transfected with 400 ng of firefly luciferase reporter plasmids, together with 25 ng Renilla luciferase construct (pRL-SV40), combined with 30 nM miR-542-5p or miR-NC mimic using Reagent (RiboBio Co, Ltd., Guangzhou, China) according to the manufacturer’s protocol. The cells were then cultured with or without TGF-β1 for another 48 h. The Renilla and firefly luciferase activities were also measured 48 h after transfection, using the Dual Luciferase Reporter Assay Kit (Promega, Madison, WI, USA). The activity of an internal firefly luciferase was normalized by Renilla luciferase activity.

### 4.11. Immunofluorescence

NIH-3T3 cells were cultured and plated in a small dish with a cover slip. After the treatment with TGF-β1 (5 ng/mL), the cells were fixed with methanol (> 99.5%) (Sinopharm Chemistry Reagent Co,. Ltd., Beijing, China) and washed with phosphate buffer solution containing 0.1% tween-20 (PBST, three times, 5 min each) and then blocked with 5% BSA (60 min). For Collagen I αI staining, cells were incubated with Collagen I α1 primary antibody (ab124964, 1:500, Abcam) at 4 °C overnight. The next day, the cells were washed with PBST (3 times, 5 min each) and then stained with the anti-rabbit secondary antibody (60 min) and 0.1% DAPI (15 min) in the dark. Finally, cells were washed with glycerol buffer and imaged with a fluoroscope (Olympus, Tokyo, Japan).

### 4.12. Cell. Cycle Assays

The NIH-3T3 cells were washed with phosphate-buffered saline and fixed with pre-chilled 75% alcohol at −20 °C, overnight. After stained with propidium iodide (PI) (5 μg/mL) for 30 min in the dark, the cells were subsequently analyzed and quantified via flow cytometry (FACSCalibur, Becton-Dickinson, Franklin Lake, NJ, USA).

### 4.13. Statistical Analysis

All data were present as the means ± SD of at least three independent experiments and representative data were reported. Data comparisons were performed by one-way analysis of variance (ANOVA) for more groups with Dunnett’s test (none treatment as the control group). SPSS 20.0 software (Armonk, NY, USA) was used for data processing and statistical analysis. A value of *p* < 0.05 was considered to be statistically significant.

## 5. Conclusions

The present study and others, suggested that integrin-mediated fibroblast activation was an important mechanism in the development of pulmonary fibrosis. Both our preventive and therapeutic mouse models showed that the ectopic expression of miR-542-5p visually attenuated a series of fibrotic lesions, including alveolar structural damage, alveolar interstitial thickening and silicon nodule formation in vivo. This indicated that miR-542-5p had an anti-fibrotic effect, not only on the acute inflammatory phase but also on the established lung fibrosis. In addition, we also identified that miR-542-5p functioned by directly binding to Itga6, the lack of which not only inhibited fibroblast activation but also reduced the phosphorylation levels of FAK/PI3K/AKT in vitro. This finding provided potential intervention targets for patients with pulmonary fibrosis, especially silicosis.

## Figures and Tables

**Table 1:** Preventive effect of miR-542-5p agomir administration on lung histopathology.

Groups	Lesion Severity Grade						Average Severity Grade	Lesion Distribution Grade						Average Severity Grade
	0	1	2	3	4	5		0	1	2	3	4	5	
Control	8							8						
SiO_2_		4	2	2			1.75 ± 0.89		6	2				1.25 ± 0.46
SiO_2_ + miR-NC		4	1	2	1		2.12 ± 1.46		4	3		1		1.75 ± 1.04
SiO_2_ + miR-542-5p	4	4					0.62 ± 0.52 *	3	4	1				0.75 ± 0.71 *

Note: Values represent the means ± SD of 8 for each group, * *p* < 0.05 vs. SiO_2_ + miR-NC group.

**Table 2:** Therapeutic effect of miR-542-5p agomir administration on lung histopathology.

Groups	Lesion Severity Grade						Average Severity Grade	Lesion Distribution Grade						Average Severity Grade
	0	1	2	3	4	5		0	1	2	3	4	5	
Control	8							8						
SiO_2_	1	1	1		1	4	3.38 ± 2.07	1	2	1	4			2.00 ± 1.20
SiO_2_ + miR-NC	1		4	1		4	2.63 ± 1.69	1	1	1	2	2	1	2.75 ± 1.67
SiO_2_ + miR-542-5p	2	2	2	1	1		1.63 ± 1.41	2	3	3				1.13 ± 0.83 *

Note: Values represent the means ± SD of 8 for each group, * *p* < 0.05 vs. SiO_2_ + miR-NC group.

## Supplementary Materials

The following are available online at http://www.mdpi.com/1422-0067/19/12/3717/s1.
