# Supplementary material for: miR-542-5p Attenuates Fibroblast Activation by Targeting Integrin α6 in Silica-Induced Pulmonary Fibrosis
Source: Int J Mol Sci. 2018 Nov 22;19(12):3717. doi: 10.3390/ijms19123717 (PMC6320929; doi:10.3390/ijms19123717)
Supplement: Supplementary file 1 [file ijms-19-03717-s001.zip › ijms-370285 sp proof done/Supplementary file 1.pdf]

Supplementary Materials and Methods

Cell culture

The human embryonic lung fibroblasts (MRC-5) were purchased from the American Type Culture Collection (ATCC, Manassas, VA, USA) and maintained in the Mimimum Essential Medium containing 10% fetal calf serum, 100 U/ml penicillin and 100 μ g/ml streptomycin. MRC-5 ells were cultured in a humidified atmosphere containing 5% CO_2 at 37 °C.

Masson-trichrome staining

Mouse lung tissues were collected and fixed as described before. Masson-trichrome staining was used to observe the collagen deposition under a slice scanner. Five microscopic fields were randomly selected and the representative parts were shown.

Supplementary results

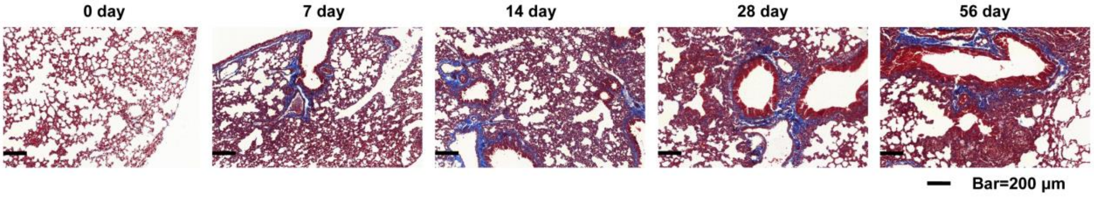

Figure S1. Masson-trichrome staining of resentative lung sections (n = 3) from each group of mice treated with silica for different times (0, 7, 14, 28 and 56 days). Increased collagen (blue) could be observed after silica treatment.

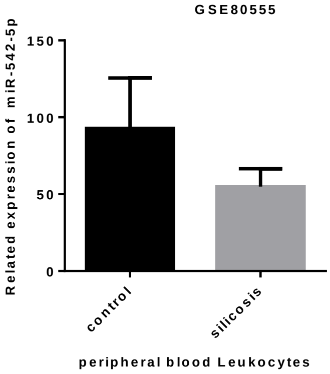

Figure S2. Relative RNA level of miR-542-5p in peripheral blood leukocytes form silicosis patients (n = 3 per group) based on the microRNA microarray dataset (GSE80555) downloarded from the Gene Expression Omnibus (GEO) database.

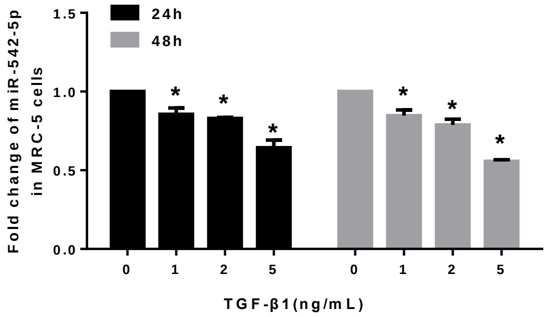

Figure S3. The miR-542-5p expression levels were significantly decreased in MRC-5 cells treated with different doses of TGF-β1 (0, 1, 2, 5 ng/mL) for 24 h or 48 h, * $p < 0.05$ versus the control group.

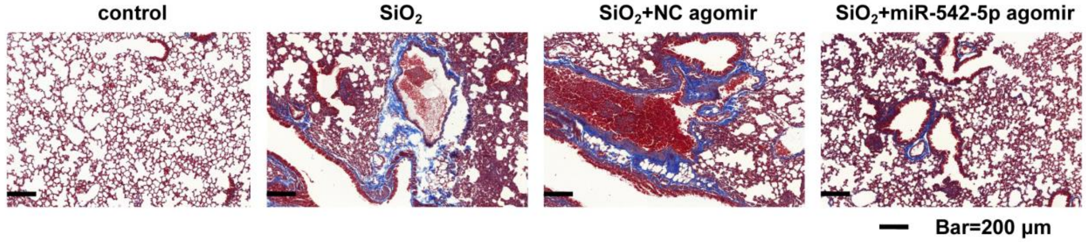

Figure S4. Masson-trichrome staining of resentative lung sections (n = 3) from each group of the prevention model. MiR-542-5p agomir effectively reduces collagen (blue) deposition caused by silica treatment.

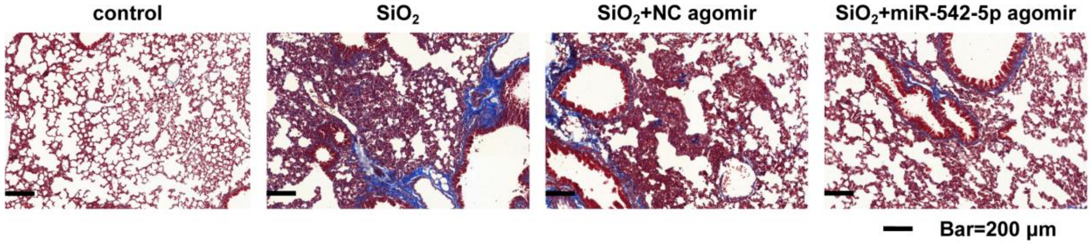

Figure S5. Masson-trichrome staining of resentative lung sections (n = 3) from each group of the treatment model. MiR-542-5p agomir exhibited a certain degree of removing the deosition of collagen (blue).
