# Supplementary material for: miR-542-5p Attenuates Fibroblast Activation by Targeting Integrin α6 in Silica-Induced Pulmonary Fibrosis
Source: Int J Mol Sci. 2018 Nov 22;19(12):3717. doi: 10.3390/ijms19123717 (PMC6320929; doi:10.3390/ijms19123717)
Supplement: Supplementary file 1 [file ijms-19-03717-s001.zip › ijms-370285 sp proof done/Supplementary file 2.pdf]

Supplemental Experimental Data

Table S1. The densitometric analysis of Western blot in Figure 1C.

	Day 1	Day 7	Day 14	Day 28	Day 56
Fibronectin	4516.104	8090.933	8574.104	11290.347	10710.347
Collagen (I)	7599.426	8420.447	15103.903	20771.539	21191.711
Vimentin	12117.711	14066.61	19110.51	21063.095	25347.61
α-SMA	7520.347	15573.104	10338.024	14350.711	13158.125
GAPDH	22381.175	22948.075	23686.761	22508.882	23291.175
Fibronectin	4674.225	8019.054	10884.882	10929.761	10160.811
Collagen (I)	15614.569	20319.397	21009.711	23386.196	30047.61
Vimentin	12976.225	17463.468	20261.054	25682.882	24055.054
α-SMA	13378.276	17883.024	26177.711	21551.782	23186.681
GAPDH	30250.317	26751.882	24531.953	21029.418	24797.66
Fibronectin	4248.376	5228.548	6178.912	7925.376	6732.255
Collagen (I)	9599.426	10420.447	15103.903	20771.539	21191.711
Vimentin	10811.326	11013.933	13532.953	17381.56	21224.903
α-SMA	18929.61	17215.075	23288.853	21651.953	26860.338
GAPDH	35607.539	33749.782	30200.418	26949.196	29613.045

Table S2. The densitometric analysis of Western blot in Figure 2D.

	Control	Silica	Silica+miR-NC	Silica+miR-542-5p
Fibronectin	10760.468	20778.731	21032.61	9207.447
Collagen (I)	10598.196	20479.045	19064.874	6708.154
Vimentin	8801.296	19879.803	17374.146	9583.853
α-SMA	7131.054	17515.024	16665.631	8070.811
GAPDH	36102.56	33205.782	29140.974	32179.368
Fibronectin	10970.69	20250.217	22556.681	10132.589
Collagen (I)	6966.154	12823.903	15073.903	5090.79
Vimentin	8836.054	20804.024	19012.61	8302.832
α-SMA	6356.669	15615.51	13600.823	6294.184
GAPDH	19133.205	20224.711	18977.296	20250.468
Fibronectin	12386.903	23956.196	23323.104	13853.983
Collagen (I)	9141.062	17385.832	17676.752	8586.66
Vimentin	9815.66	20338.731	20513.903	10265.095
α-SMA	13327.539	24303.731	22725.731	13541.539
GAPDH	23700.225	16995.175	16988.175	14781.447

Table S3. The densitometric analysis of Western blot in Figure 3D.

	Control	Silica	Silica+miR-NC	Silica+miR-542-5p
Fibronectin	13156.225	23432.125	28795.974	12543.861
Collagen (I)	12316.024	30640.238	34925.167	15922.075
Vimentin	6301.489	14033.995	12709.581	5456.619
α-SMA	12547.832	22617.167	19130.51	7207.539
GAPDH	30660.539	21629.518	19244.317	23227.359
Fibronectin	9759.447	19472.853	19022.803	10891.56
Collagen (I)	11628.581	25828.602	25603.359	10403.368
Vimentin	17700.539	26325.489	28842.116	16589.539
α-SMA	9097.196	19438.095	19735.581	10749.288
GAPDH	21128.74	22674.761	23260.761	22134.518
Fibronectin	9998.447	25651.267	23986.075	14182.418
Collagen (I)	15781.146	31573.53	28553.581	8952.69
Vimentin	20461.974	27618.56	27360.853	16581.518
α-SMA	5488.184	14836.116	13794.338	6696.953
GAPDH	19541.861	19250.397	19237.347	19103.246

Table S4. The densitometric analysis of Western blot in Figure 4A (left).

TGF-β1	0 ng/mL	1 ng/mL	2 ng/mL	5 ng/mL
Fibronectin	7209.782	13691.51	16130.631	17659.56
Collagen (I)	11316.225	15538.276	15044.74	19337.69
Vimentin	19013.489	27812.075	31232.66	35399.267
α-SMA	2959.598	6826.518	8512.983	9933.246
GAPDH	12146.338	14718.853	14694.196	14741.61
Fibronectin	6155.598	11272.66	13995.711	13986.459
Collagen (I)	11352.167	12605.903	15925.61	19016.803
Vimentin	24245.368	33590.953	37075.439	31800.489
α-SMA	4330.447	8787.276	12802.569	11415.711
GAPDH	17640.175	15635.004	16248.711	14974.004
Fibronectin	7760.874	24390.966	22484.35	23853.2
Collagen (I)	8537.832	10769.811	10390.276	18220.468
Vimentin	6558.749	8708.489	13599.811	19057.539
α-SMA	5608.355	8722.832	14526.439	19739.246
GAPDH	18476.418	18811.167	17892.924	23830.995

Table S5. The densitometric analysis of Western blot in Figure 4D (right).

TGF-β1	0 ng/mL	1 ng/mL	2 ng/mL	5 ng/mL
Fibronectin	11710.589	22382.388	24234.338	32300.217
Collagen (I)	11916.196	20543.246	26214.246	28894.075
Vimentin	4756.092	9247.054	17667.711	17860.631

α-SMA	12401.418	17188.288	20568.995	26169.238
GAPDH	21518.024	21655.489	20709.388	24849.267
Fibronectin	13220.974	25971.803	24222.267	24353.196
Collagen (I)	8974.669	19452.631	20055.56	25212.459
Vimentin	19834.024	22918.066	24067.874	33848.966
α-SMA	7738.418	13750.874	20451.752	27348.995
GAPDH	13715.376	14030.861	15552.64	16507.841
Fibronectin	10969.66	24885.874	22621.752	28116.652
Collagen (I)	6813.82	8216.882	10892.338	15425.075
Vimentin	12633.539	19867.681	25806.439	30257.368
α-SMA	8820.69	15331.016	20137.673	28444.108
GAPDH	20180.296	20891.69	17375.761	17380.66

Table S6. The densitometric analysis of Western blot in Figure 4D.

	Control	TGF-β1	TGF-β1+miR-NC	TGF-β1+miR-542-5p
Fibronectin	6041.054	18123.56	15634.61	9028.539
Collagen (I)	3458.447	8367.196	10911.167	3925.569
Vimentin	6847.205	12184.619	11054.276	3324.033
α-SMA	5389.439	11454.489	14401.974	4088.569
GAPDH	16063.874	19876.56	19336.439	20538.146
Fibronectin	8380.125	14220.924	12469.095	9617.347
Collagen (I)	6954.054	10742.217	10042.823	5071.368
Vimentin	4110.033	9784.761	11291.61	6222.296
α-SMA	3507.569	7423.569	7716.811	2811.669
GAPDH	14540.054	13035.196	14851.61	15369.075
Fibronectin	3704.841	10855.882	11247.439	6215.296
Collagen (I)	3785.66	9764.146	8208.903	4719.388
Vimentin	5761.548	10700.832	12703.539	7347.882
α-SMA	4862.083	12862.004	14480.246	7451.347
GAPDH	17386.782	18929.196	15333.347	16188.832

Table S7. The densitometric analysis of Western blot in Figure 5D.

	Control	TGF-β1	TGF-β1+miR-NC	TGF-β1+miR-542-5p
ITGA6	11234.317	21810.459	18426.045	12304.267
GAPDH	21187.146	25129.61	19714.075	22688.317
ITGA6	10623.234	22281.924	26306.388	16969.711
GAPDH	34728.631	25171.095	30233.024	29562.075
ITGA6	14965.075	26859.731	24830.075	17678.054

GAPDH	19286.468	26284.439	22556.347	18728.811

Table S8. The densitometric analysis of Western blot in Figure 7B.

	Control	TGF-β1	TGF-β1+vector	TGF-β1+Itga6
ITGA6	3459.184	24565.146	23980.167	4466.447
Fibronectin	10087.397	14231.196	19859.782	13022.439
Collagen (I)	7292.933	10916.095	12324.024	8446.539
Vimentin	12255.782	16785.66	15861.832	10446.175
α-SMA	7780.539	25189.773	19874.652	10626.539
GAPDH	17493.589	16012.903	18628.489	19329.347
ITGA6	5002.083	18786.125	10543.024	2473.577
Fibronectin	8074.69	12016.255	10995.477	7684.134
Collagen (I)	19883.61	27995.631	28262.874	20635.903
Vimentin	7741.225	15627.51	13569.995	7963.811
α-SMA	21993.276	32298.995	28906.995	14950.134
GAPDH	7193.054	5787.811	5885.518	5661.811
ITGA6	5576.154	20523.075	25679.933	6026.125
Fibronectin	5867.983	11031.347	12005.539	9892.225
Collagen (I)	8567.368	21616.459	13982.631	5581.175
Vimentin	4488.861	13579.489	13041.882	9457.711
α-SMA	8173.782	18977.054	16684.539	4264.397
GAPDH	11170.861	11642.397	11424.104	14109.64

Table S9. The densitometric analysis of Western blot in Figure 7C.

	Control	TGF-β1	TGF-β1+vector	TGF-β1+Itga6
P-FAK	5111.61	15988.581	17140.238	7445.773
FAK	23464.539	24637.711	27502.296	20014.267
P-PI3K	25217.459	35205.995	27948.924	12081.066
PI3K	18547.267	21237.924	25066.439	25940.924
P-AKT	2882.284	16196.146	21233.823	8383.803
AKT	19858.983	18204.74	21116.953	16844.104
GAPDH	25695.823	20105.903	21709.368	20548.731
P-FAK	17453.539	24738.095	25400.924	12201.054
FAK	18555.539	24694.388	28113.217	32464.51
P-PI3K	10997.267	14956.167	15105.853	9890.61
PI3K	21015.731	21172.146	25745.782	30942.681
P-AKT	3730.083	8306.74	6183.326	2870.134
AKT	17407.468	17243.004	14419.497	16014.882
GAPDH	20614.983	16533.711	17892.196	18687.64
P-FAK	7579.61	14298.924	13813.924	4026.903
FAK	16962.024	17465.024	17977.296	19534.903
P-PI3K	10906.823	20726.288	15952.874	5534.589

PI3K	11201.761	12579.054	12988.933	11164.64
P-AKT	4099.042	16416.711	10416.861	3514.234
AKT	18819.761	15662.669	17130.841	19418.64
GAPDH	24465.075	29933.054	31601.004	28374.439

Table S10. The densitometric analysis of Western blot in Figure 7D.

	Control	TGF-β1	TGF-β1+miR-NC	TGF-β1+miR-542-5p	TGF-β1+miR-542-5p+vector	TGF-β1+miR-542-5p+ITGA6
ITGA6	6149.882	14130.681	10156.861	4027.912	4135.912	10995.933
Fibronectin	14371.276	23246.468	24871.225	15446.64	15080.64	24863.953
Collagen (I)	13772.225	18059.79	18211.962	12868.426	10987.518	15458.104
Vimentin	7303.711	14338.539	12289.225	8811.933	6515.882	12181.953
α-SMA	10162.933	14826.083	13241.74	8418.74	9284.154	15539.882
GAPDH	19924.711	18704.962	17709.397	17575.154	18440.497	17330.075
ITGA6	5820.033	14278.004	11493.711	3767.548	3219.912	14255.731
Fibronectin	7232.175	18034.296	16674.711	7743.397	6635.397	9582.589
Collagen (I)	15881.569	25653.811	25627.296	13477.225	12794.711	22021.853
Vimentin	8474.426	13056.326	9354.983	4281.79	6084.74	13111.64
α-SMA	4864.74	17125.024	14544.347	3864.134	3110.234	11897.054
GAPDH	15761.317	16076.033	15612.125	15004.418	16688.811	16843.497
ITGA6	4411.234	13434.296	17564.882	5809.276	5098.326	14475.933
Fibronectin	13913.903	20783.296	17031.296	10385.619	9817.69	14640.296
Collagen (I)	13952.347	28488.953	25025.468	17897.882	16825.347	27646.054
Vimentin	9876.74	13749.569	14985.154	9776.912	6380.991	18924.66
α-SMA	4068.255	10755.953	10591.196	2266.598	2045.406	12819.957
GAPDH	10906.104	11828.912	10265.619	10900.69	11350.69	12557.681
