# Supplementary figures and images for: miR-542-5p Attenuates Fibroblast Activation by Targeting Integrin α6 in Silica-Induced Pulmonary Fibrosis
Source: Int J Mol Sci. 2018 Nov 22;19(12):3717. doi: 10.3390/ijms19123717 (PMC6320929; doi:10.3390/ijms19123717)

Figure 1C

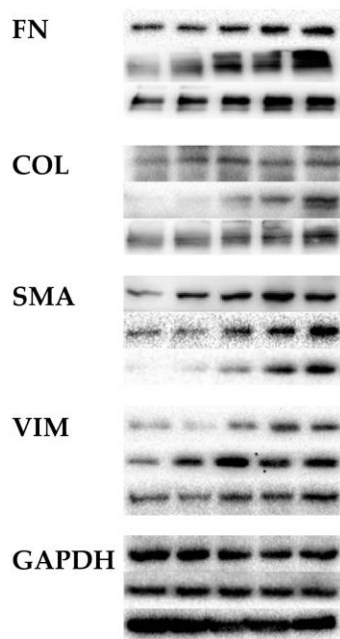

Figure 2D

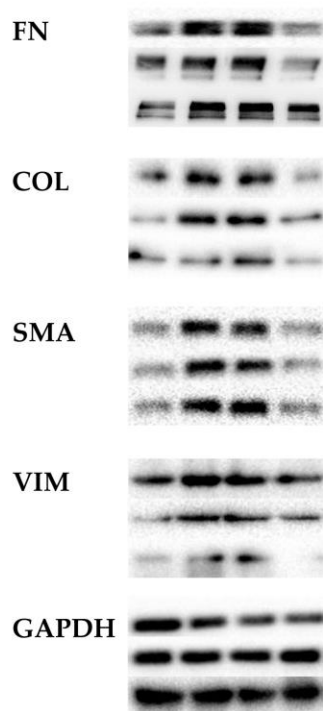

Figure 3D

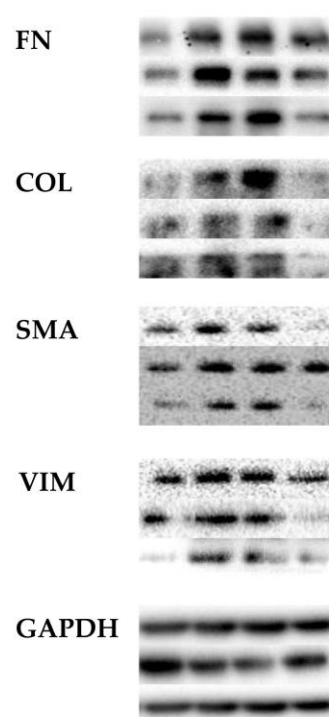

Figure 4A (left)

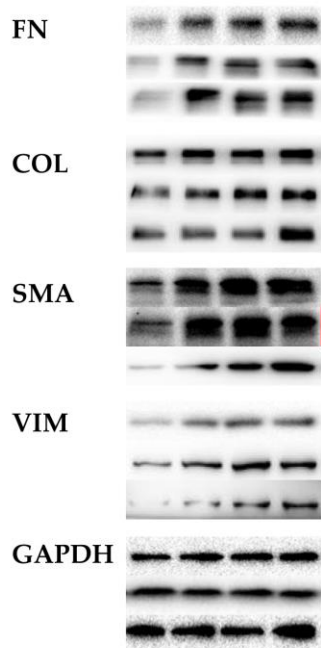

Figure 4A (right)

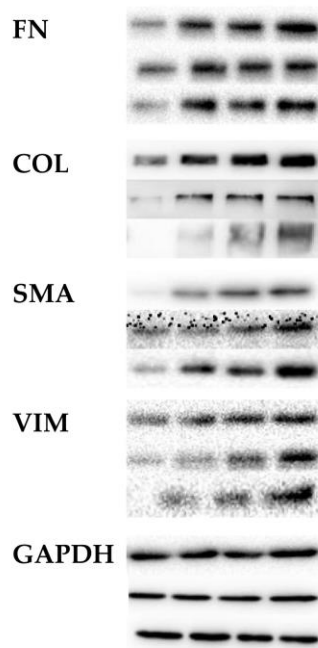

Figure 4D

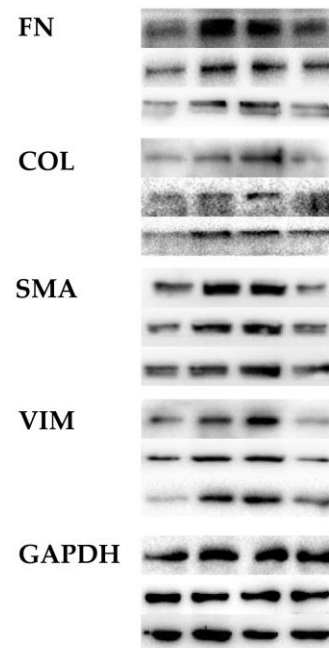

Figure 6D

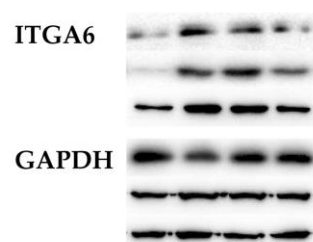

Figure 7B

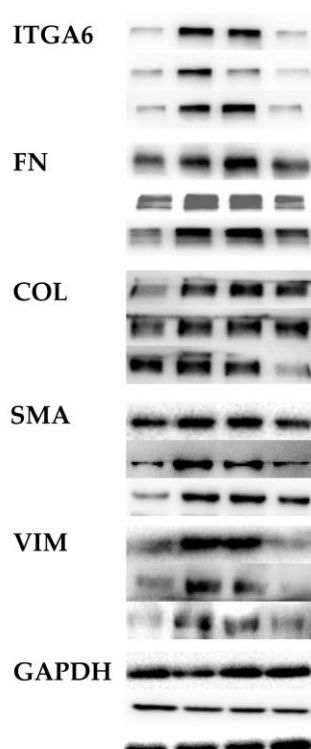

Figure 7C

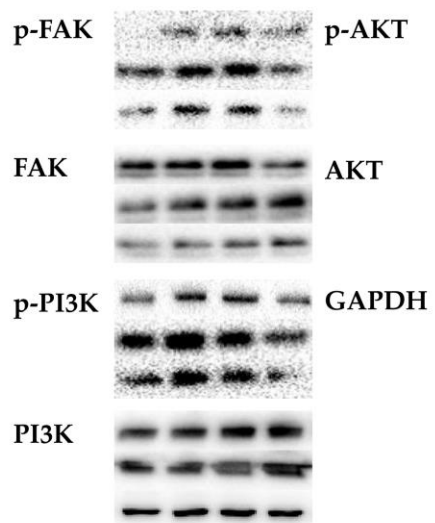

Figure 7D

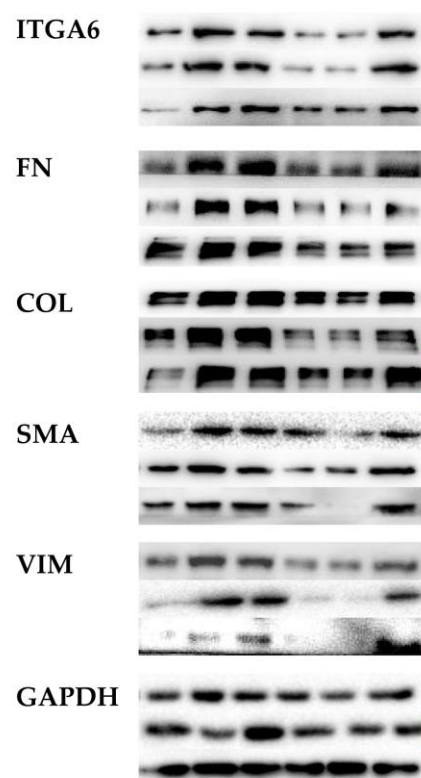

Supplement: Supplementary file 1 [file ijms-19-03717-s001.zip › ijms-370285 sp proof done/Supplementary file 3.pdf]
